# A hypoperfusion context may aid to interpret hyperlactatemia in sepsis-3 septic shock patients: a proof-of-concept study

**DOI:** 10.1186/s13613-017-0253-x

**Published:** 2017-03-09

**Authors:** Leyla Alegría, Magdalena Vera, Jorge Dreyse, Ricardo Castro, David Carpio, Carolina Henriquez, Daniela Gajardo, Sebastian Bravo, Felipe Araneda, Eduardo Kattan, Pedro Torres, Gustavo Ospina-Tascón, Jean-Louis Teboul, Jan Bakker, Glenn Hernández

**Affiliations:** 1 Departamento de Medicina Intensiva, Facultad de Medicina, Pontificia Universidad Católica de Chile, Marcoleta 367, Santiago, 8320000 Chile; 2 Hospital San Francisco, Quito, Ecuador; 3 Intensive Care Medicine Department, Fundación Valle del Lili - Universidad ICESI, Cali, Colombia; 4 Assistance Publique-Hôpitaux de Paris, Hôpitaux universitaires Paris-Sud, Hôpital de Bicêtre, Service de Réanimation Médicale, Le Kremlin-Bicêtre, France; 5 Department of Intensive Care Adults, Erasmus University Medical Center, Rotterdam, The Netherlands

**Keywords:** Septic shock, Hyperlactatemia, Resuscitation, Hypoperfusion, Central venous oxygen saturation, Central venous-arterial PCO_2_ gradient, Capillary refill time

## Abstract

**Background:**

Persistent hyperlactatemia is particularly difficult to interpret in septic shock. Besides hypoperfusion, adrenergic-driven lactate production and impaired lactate clearance are important contributors. However, clinical recognition of different sources of hyperlactatemia is unfortunately not a common practice and patients are treated with the same strategy despite the risk of over-resuscitation in some. Indeed, pursuing additional resuscitation in non-hypoperfusion-related cases might lead to the toxicity of fluid overload and vasoactive drugs. We hypothesized that two different clinical patterns can be recognized in septic shock patients through a multimodal perfusion monitoring. Hyperlactatemic patients with a hypoperfusion context probably represent a more severe acute circulatory dysfunction, and the absence of a hypoperfusion context is eventually associated with a good outcome. We performed a retrospective analysis of a database of septic shock patients with persistent hyperlactatemia after initial resuscitation.

**Results:**

We defined hypoperfusion context by the presence of a ScvO_2_ < 70%, or a P(cv-a)CO_2_ ≥6 mmHg, or a CRT ≥4 s together with hyperlactatemia. Ninety patients were included, of whom seventy exhibited a hypoperfusion-related pattern and 20 did not. Although lactate values were comparable at baseline (4.8 ± 2.8 vs. 4.7 ± 3.7 mmol/L), patients with a hypoperfusion context exhibited a more severe circulatory dysfunction with higher vasopressor requirements, and a trend to longer mechanical ventilation days, ICU stay, and more rescue therapies. Only one of the 20 hyperlactatemic patients without a hypoperfusion context died (5%) compared to 11 of the 70 with hypoperfusion-related hyperlactatemia (16%).

**Conclusions:**

Two different clinical patterns among hyperlactatemic septic shock patients may be identified according to hypoperfusion context. Patients with hyperlactatemia plus low ScvO_2_, or high P(cv-a)CO_2_, or high CRT values exhibited a more severe circulatory dysfunction. This provides a starting point to launch further prospective studies to confirm if this approach can lead to a more selective resuscitation strategy.

**Electronic supplementary material:**

The online version of this article (doi:10.1186/s13613-017-0253-x) contains supplementary material, which is available to authorized users.

## Background

Sepsis-3 consensus defined septic shock as vasopressor requirement to maintain mean arterial pressure (MAP) >65 mmHg and a serum lactate level >2 mmol/L despite fluid resuscitation [1], and claimed that these criteria will identify patients with a homogeneous high risk of death, allegedly in excess of 40% [1]. Unfortunately, neither this new definition [1], or the Surviving Sepsis Campaign (SSC) [2] recognizes the heterogeneous sources of lactate in septic shock or the existence of different clinical patterns among hyperlactatemic patients. Therefore, normalization of lactate is recommended as a goal for septic shock resuscitation under the assumption that tissue hypoxia is the main source of lactate generation [2].

However, persistent hyperlactatemia is particularly difficult to interpret in the clinical setting. Besides hypoperfusion, adrenergic-driven aerobic lactate production and impaired hepatic lactate clearance have been suggested as important contributors [3–5]. Recognizing a clinical pattern of hypoperfusion-related hyperlactatemia appears as highly relevant since optimizing systemic blood flow in this setting could revert ongoing hypoperfusion and improve prognosis [6, 7]. In contrast, pursuing additional resuscitation in non-hypoperfusion-related cases might lead to the toxicity of fluid overload and excessive vasoactive drugs, eventually increasing morbidity or mortality [4].

Recognition of clinical patterns among septic shock patients with persistent hyperlactatemia is unfortunately not a common practice around the world, and hyperlactatemic patients are treated with the same strategy despite the risk of over-resuscitation in some. Nevertheless, a multimodal perfusion monitoring could aid in identifying a hypoperfusion context [4]. Indeed, the combination of hyperlactatemia with pCO_2_-related markers of tissue hypoxia provides a stronger prognostic signal than either one alone [8]. In a lactate-driven resuscitation protocol, Jansen et al. [7] recommended systemic flow optimization exclusively in hyperlactatemic patients with concomitant low central venous oxygen saturation (ScvO_2_). Hernandez et al. [9] demonstrated that variables such as ScvO_2_, central venous-arterial PCO_2_ gradient (P(cv-a)CO_2_) and peripheral perfusion as assessed by the capillary refill time (CRT) exhibit a very fast normalization rate in relation to systemic flow optimization, whereas lactate shows a biphasic response with an initial rapid improvement in parallel with the above-mentioned variables, followed by a much slower trend thereafter. Thus, a concomitant low ScvO_2_, or high P(cv-a)CO_2_, or an abnormal peripheral perfusion defines a hypoperfusion context on which increasing systemic flow may contribute to serum lactate decrease.

Is the opposite true? Can resuscitation be safely stopped in hyperlactatemic septic shock patients without a hypoperfusion context? A recent pilot study by van Genderen et al. [10] indeed suggests that stopping fluid resuscitation when peripheral perfusion is normalized is not only safe, but also associated with less positive fluid balances and organ dysfunctions in septic shock patients with hyperlactatemia. However, this aspect is currently being addressed by ongoing studies.

We hypothesized that two different clinical patterns can be recognized in septic shock patients as defined by sepsis-3 consensus, according to the presence of a hypoperfusion context. Hyperlactatemic patients with a hypoperfusion context probably represent a more severe acute circulatory dysfunction, and the absence of a hypoperfusion context is eventually associated with a good outcome. We designed this retrospective proof-of-concept study to test our primary hypothesis. If this proves to be true, then it could be safe to launch a prospective study testing a restrictive resuscitation strategy in the latter, while concentrating resuscitation efforts in the former.

## Methods

We evaluated all septic shock patients admitted to our intensive care unit (ICU) at the University Hospital of the Pontificia Universidad Católica de Chile during the 18-month period from July 2013 to December 2014. These patients were managed according to a local algorithm [9] and entered into a prospective dataset. The Institutional Review Board of our Institution approved this study and waived the need of an informed consent because of the retrospective observational nature of the study (CEC-MEDUC; 16-182, 2016).

### Study population

Patients ≥18 years old were included if they presented: (a) septic shock according to sepsis-3 Definitions (requirement of vasopressors to sustain MAP ≥65 mmHg and hyperlactatemia >2 mmol/l after initial fluid resuscitation) [1]; (b) concomitant values for ScvO_2_, P(cv-a)CO_2_ and CRT; (c) full commitment for resuscitation; and (d) complete follow-up in the dataset and medical charts until hospital discharge.

### Study protocol

Hypoperfusion context was defined by the presence of a ScvO_2_ <70%, or a *P*(cv-a) CO_2_ ≥6 mmHg, or a CRT ≥4 s together with hyperlactatemia after initial fluid resuscitation in the ICU. Patients without any abnormality were classified as with a non-hypoperfusion context. Both subgroups were compared in clinical-demographic data and severity scores at baseline; and macrohemodynamic and perfusion parameters, hemodynamic interventions and main outcomes.

All patients were managed according to our local algorithm aimed at macrohemodynamic stabilization and improvement of hypoperfusion [9]. A central venous catheter and an arterial line were inserted in all. A pulmonary artery catheter was recommended for those with a concomitant acute respiratory distress syndrome and/or past medical history of heart failure. Perfusion targets were normalization of ScvO_2_, P(cv-a)CO_2_, CRT and mottling, and a clear tendency to a decrease in arterial lactate levels. The algorithm was described elsewhere [9], but briefly preload optimization guided by dynamic predictors of fluid responsiveness was the first step to improve systemic blood flow. Pulse pressure variation was the preferred dynamic parameter when feasible. Echocardiography was not routinely performed during the study period except as an aid to assess preload status through inferior vena cava distensibility when necessary. Fluid challenges were repeated until the perfusion targets mentioned above were normalized, or the selected dynamic predictor of fluid responsiveness turned negative, or a safety limit of an increase in central venous pressure ≥5 mmHg after a fluid bolus was reached. Norepinephrine (NE) was the vasopressor of choice and adjusted to a MAP ≥65 mmHg. Low-dose dobutamine or milrinone was restricted to patients with persistent or worsening hypoperfusion after preload optimization. Hemoglobin concentrations were maintained at 8 g/dL or higher to optimize arterial O_2_ content. Mechanical ventilation settings were adjusted according to current recommendations. High-volume hemofiltration (HVHF) was used as rescue therapy in patients evolving with refractory septic shock.

### Statistical analysis

Categorical data were analyzed with Chi-square, *t* test for paired samples and Wilcoxon test when appropriate. We tested the discriminating value of each perfusion variable alone or in combination.

All data are presented as mean ± standard deviation (SD). All reported p values are two-sided, with a significant alpha level at 5%. STATA 13 (STATA Corp, Tx, USA) statistical package was used for analysis.

## Results

A total of 116 septic shock patients were admitted during the study period. Of these, ninety patients (57% female, mean age 66 ± 16 years) fulfilling the inclusion criteria were analyzed. Twenty-six patients were excluded from further analysis because of normal lactate levels at ICU admission (7), absence of data on one of the three parameters that define hypoperfusion context (10), no full commitment for full resuscitation (4), or early transfer to other hospitals (5).

The main sepsis sources were abdominal (*n* = 46), pulmonary (*n* = 21), urinary (*n* = 15), and others (*n* = 8). Seventy patients exhibited a hypoperfusion context after initial resuscitation in the ICU, and 20 did not. The main clinical, demographic and physiological characteristics according to subgroups at baseline are presented in Table 1.

**Table 1:** Clinical, demographic, severity scores, perfusion and hemodynamic variables at baseline according to hypoperfusion context

	Hypoperfusion context (70)	Non-hypoperfusion context (20)	p value
Age (years)	66 ± 17	65 ± 13	0.9
Charlson index	1.9 ± 2.2	1.6 ± 0.3	0.7
APACHE II score	22 ± 7	20 ± 5	0.2
SOFA score	9.5 ± 3.8	8.4 ± 2.6	0.1
Heart rate (beats/s)	104 ± 23	115 ± 24	0.07
MAP (mmHg)	75 ± 19	77 ± 21	0.6
SAP (mmHg)	108 ± 27	111 ± 27	0.6
DAP (mmHg)	59 ± 14	57 ± 12	0.6
Arterial lactate (mmol/L)	4.8 ± 2.8	4.7 ± 3.7	0.9
ScvO2 (%)	71.3 ± 9.5	74.8 ± 7	<0.05
*P*(cv-a) CO_2_	7.6 ± 2.5	5.5 ± 2.2	<0.001
CRT (s)	5.4 ± 2.3	4.2 ± 2.6	<0.001
NE (mcg/kg/min)	0.19 ± 0.24	0.09 ± 0.11	<0.05

A p < 0.05 was considered as significant

Values are expressed as mean ± SD

*APACHE* Acute Physiology and Chronic Health Evaluation, *SOFA* Sequential Organ Failure Assessment, *MAP* mean arterial pressure, *SAP* systolic arterial pressure, *DAP* diastolic arterial pressure, *P*(cv-a)*CO*_*2*_ central venous-arterial pCO_2_ gradient, *CRT* capillary refill time, *NE* norepinephrine

Although lactate values at baseline (4.8 ± 2.8 vs. 4.7 ± 3.7 mmol/L) were comparable, the hypoperfusion-context subgroup presented a more severe circulatory dysfunction as expressed by higher NE requirements and a trend to a worse SOFA score (Table 1). As expected according to our local algorithm, patients categorized as with a hypoperfusion context received more dobutamine (31 vs. 5%; p < 0.01), and tended to receive more fluids (6732 ± 2524 vs. 5940 ± 2756 ml; p = 0.2).

Lactate decreased at the same rate in both groups (6 h lactate: 4 ± 2.7 vs. 3.9 ± 2.7 mmol/l, p = 0.9; 24 h lactate: 3.2 ± 3.0 vs. 2.7 ± 2.1 mmol/l, p = 0.4; 6 h lactate clearance: 13 ± 39 vs. 7 ± 48%, p = 0.58; 24 h lactate clearance: 27 ± 61 vs. 32 ± 39%, p = 0.7). However, within the hypoperfusion-context subgroup, 24-h lactate levels significantly decreased only in survivors with no change in non-survivors (Additional file 1: Figure S1). In addition, P(cv-a)CO_2_ (7.6 ± 2.6 to 4.4 ± 2.3 mmHg; p < 0.001) and CRT (5.4 ± 2.2 to 3.1 ± 2.9 s; p < 0.001) decreased only in survivors.

**Fig. 1:**
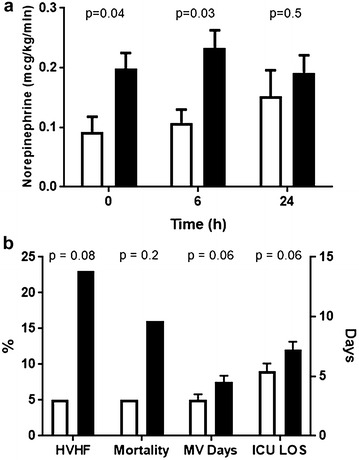
Severity criteria to compare hyperlactatemic patients without versus with a hypoperfusion context. **a** Mean norepinephrine doses for both subgroups at baseline, 6 and 24 h. *Black boxes* describe hypoperfusion-context subgroup; *white boxes* represent non-hypoperfusion context subgroup. **b** Comparison of the use of rescue therapy and several outcome parameters between subgroups. *HVHF* high-volume hemofiltration, *Mortality* hospital mortality, *MV* mechanical ventilation, *ICU* intensive care unit, *LOS* length of stay

Regarding main outcomes, patients with a hypoperfusion context exhibited a more severe circulatory dysfunction requiring higher NE doses at 6 h, and tended to have longer duration of mechanical ventilation, and higher use of HVHF as rescue therapy, in addition to longer ICU and hospital stays (Fig. 1). Eleven of 70 patients (16%) with a hypoperfusion context died as compared to only one of twenty in those without, although this difference was not significant.

**Fig. 2:**
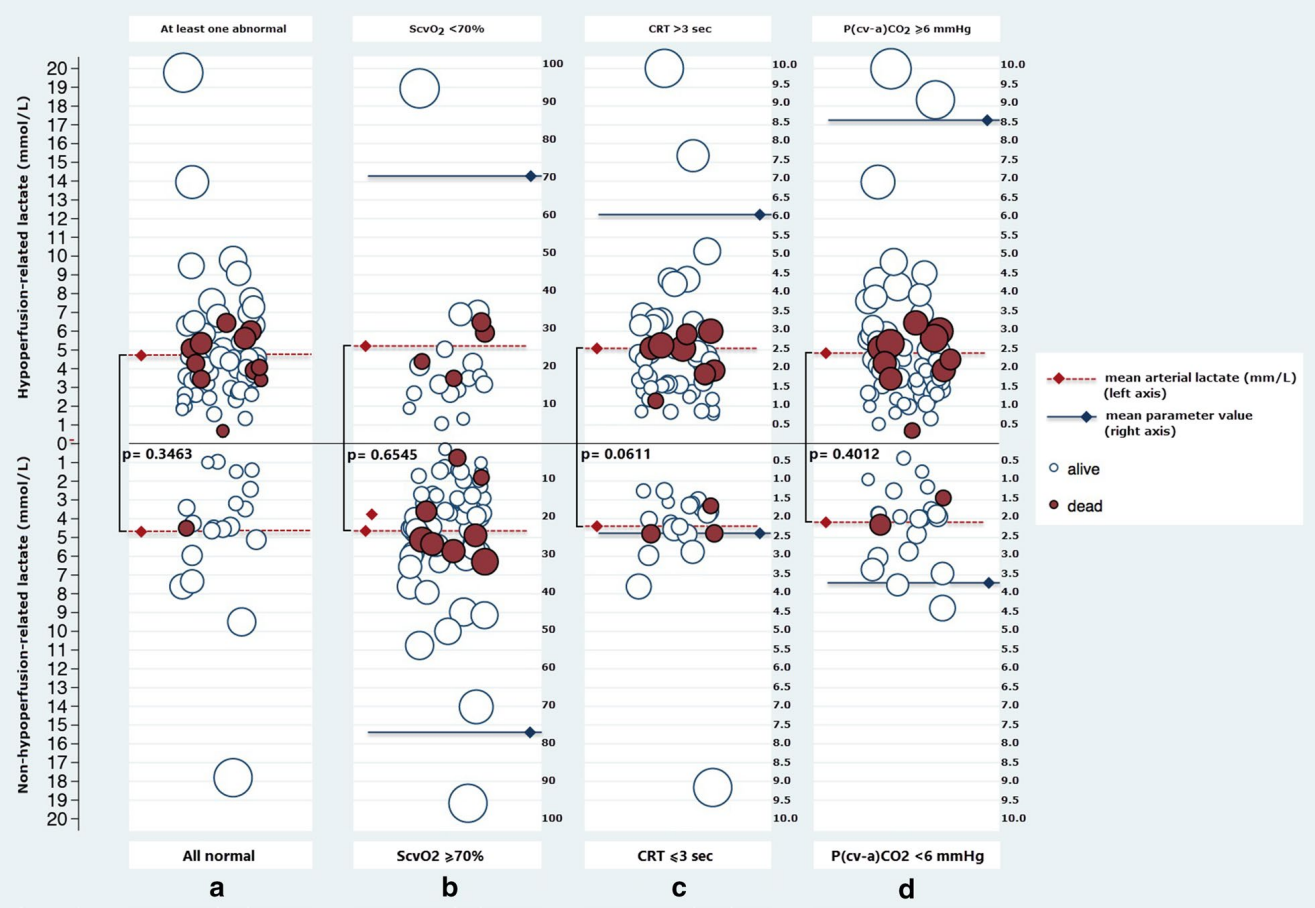
Distributional figure: the figure displays different plausible allocation of patients under *four* distinctive hypoperfusion-context descriptors and their relationship with outcome in hyperlactatemic septic shock patients. The figure shows the distribution of patients according to the presence of abnormal ScvO_2_, CRT, and P(cv-a)CO_2_, and its relationship with hospital mortality where *white* and *black circles* represent survivors and non-survivors, respectively. **a** Comparing patients with normal perfusion criteria (*bottom*) versus those with at least one abnormal criterion (*top*). **b** Comparing patients with normal (*bottom*) versus abnormal ScvO_2_ (*top*). **c** Comparing patients with normal (*bottom*) versus abnormal CRT (*top*). **d** Comparing patients with normal (*bottom*) versus abnormal P(cv-a)CO_2_ (*top*). The *p* values represent the difference in mean lactate values among patients fulfilling or not the descriptors tested. No difference in lactate values was observed when using any of these descriptors although there are trends for difference in survival, especially when using the “at least one abnormal perfusion parameter” criterion as shown in the *first column*. *ScvO*_*2*_ central venous oxygen saturation, *CRT* capillary refill time, *P*(cv-a)*CO*_*2*_ central venous-to-arterial carbon dioxide difference

The effect of using the criterion of “at least one abnormal perfusion parameter” among the three considered for defining a hypoperfusion context, as compared to each one individually to discriminate survivors versus non-survivors is shown in the distributional Fig. 2. Although the purpose of this figure is only to show visually the impact of using different criteria, it appears that the best discriminating capacity is for “at least one abnormal perfusion parameter.” In addition, the effect of testing different combinations of these three perfusion parameters (ScvO_2_, P(cv-a)CO_2_, and CRT) to define hypoperfusion context and the effect on outcomes variables is shown in Additional file 2: Table S1.

## Discussion

Our main finding is that a multimodal perfusion monitoring in sepsis-3 septic shock patients may aid to differentiate two clinical patterns according to the presence of a hypoperfusion context. Patients with hyperlactatemia associated with either low ScvO_2_, or high P(cv-a)CO_2_, or high CRT values exhibit a more severe circulatory dysfunction. The absence of a hypoperfusion context among septic shock patients with hyperlactatemia is associated with a good outcome. These findings do not support sepsis-3 statement that the new septic shock definition identifies a group of patients with a homogenous high mortality risk [1]. On the other hand, they provide the basis for the development of a prospective study aimed at testing a safe restrictive fluid resuscitation strategy in septic shock patients without a hypoperfusion context, while concentrating resuscitation efforts in those with hypoperfusion criteria. The presence of a low ScvO_2_ in the setting of persistent hyperlactatemia clearly indicates an imbalance in the oxygen transport/oxygen consumption relationship [7, 11]. A high Pcv–aCO_2_ might identify patients who remain inadequately resuscitated and constitutes a marker of global perfusion that could aid in identifying a hypoperfusion-related hyperlactatemia [12, 13]. Assessment of peripheral perfusion may provide additional physiological information. An abnormal peripheral perfusion may be caused by adrenergic-induced skin vasoconstriction secondary to a low systemic blood flow and should prompt a reassessment of the hemodynamic state, in particular the volume status [10, 14]. Under the best of our knowledge, this is the first study that uses the three variables together to define a hypoperfusion context, an aspect that has to be highlighted because of the individual limitations of every isolated parameter. As a matter of fact, ScvO_2_ can be normal in the setting of severe microcirculatory abnormalities [4, 12], Pcv-aCO_2_ might be normal in hyperdynamic states even in the presence of tissue hypoxia [13], and peripheral perfusion may be difficult to assess under some circumstances. However, our results show that when the three are normal in hyperlactatemic patients, mortality as well as morbidity is very low, and thus the presence of tissue hypoxia as the cause of hyperlactatemia appear as unlikely in this setting, although of course it cannot be ruled out completely.

Remarkably, lactate values were comparable between subgroups at baseline (mean of 4.7–4.8 mmol/l) at a level where patients would not only have been diagnosed as septic shock according to sepsis-3 criteria [1], but also considered for inclusion in the EDGT trial [11]. However, adding hypoperfusion-context criteria to clinical assessment might aid in differentiating two clinical patterns with marked different risks as demonstrated by our study. Of course our design does not allow us to establish sensitivity or specificity or predictive value of these criteria to track real hypoxia-related hyperlactatemia, but at least our findings are hypothesis-generating, and might build up the foundations for future trials on septic shock patients that focus resuscitation efforts on selected populations. Anyway, hypoxia is a cellular event that is hardly diagnosed by clinical means. In the past, lactate/pyruvate ratio or more recently, the venous-arterial CO_2_ to arterial-venous O_2_ content difference ratio have been proposed for this purpose, although both ratios have important theoretical and practical drawbacks [3, 8]. Our study has several drawbacks related to its retrospective nature and inclusion of a relatively small number of patients. Several variables exhibited a trend to be worse in the hypoperfusion-related subgroup but were short of significance, thus weakening our conclusions. Additionally, each variable proposed to define a hypoperfusion context has its own limitations. We also did not calculate the lactate/pyruvate or venous-arterial CO_2_ to arterial-venous O_2_ content difference ratios, closers indicators of tissue hypoxia according to several physiological studies. The parallel decrease in lactate levels in subgroups with versus without a hypoperfusion context could be viewed as contradictory, but when addressing this aspect more profoundly, the rate of decrease in lactate values in presumably hypoperfused patients was slowed by the performance of non-survivors who tended to increase lactate over time as compared to survivors (Additional file 1: Figure S1). This highlights the difficulties in interpreting lactate dynamics in septic shock patients subjected to a nonselective resuscitation strategy, as was also observed in another previous study [7]. However, despite these limitations, we could effectively differentiate two clinical patterns among hyperlactatemic septic shock patients when applying a monitoring model that includes three easily assessable perfusion-related parameters.

## Conclusion

A mutimodal perfusion monitoring can identify two clinical patterns among hyperlactatemic septic shock patients according to widely used hypoperfusion-related criteria. Patients with hyperlactatemia plus low ScvO_2_, or high P(cv-a)CO_2_, or high CRT values exhibit a more severe circulatory dysfunction with increased morbidity. This provides a starting point to launch further prospective studies to confirm if this approach can lead to a more rational and selective resuscitation strategy.

## Additional files

Additional file 1.
**Figure S1** Evolution of serum lactate levels over time (0, 6 and 24h) in different subgroups: patients without hypoperfusion context, and survivors and non-survivors in the hypoperfusion-context subgroup.
Additional file 2.
**Table S1** Clinical, demographic, severity scores, perfusion and hemodynamic variables at baseline for the whole cohort and according to different combinations of hypoperfusion criteria. A p < 0.05 was considered as significant. Values are expressed as mean ± SD. *APACHE* Acute Physiology and Chronic Health Evaluation, *SOFA* Sequential Organ Failure Assessment, *P(cv-a)CO*_2_ central venous-arterial pCO_2_ gradient, *ICU* intensive care unit, *LOS* length of stay.

## Abbreviations

MAP: mean arterial pressure
ScvO2: central venous oxygen saturation
P(cv-a)CO_2_: central venous-arterial PCO_2_ gradient
SSC: Surviving Sepsis Campaign
CRT: capillary refill time
ICU: intensive care unit

## References

[1] Singer M, Deutschman CS, Seymour CW, Shankar-Hari M, Annane D, Bauer M (2016). The third international consensus definitions for sepsis and septic shock (Sepsis-3). JAMA.

[2] Dellinger RP, Levy MM, Rhodes A, Annane D, Gerlach H, Opal SM (2013). Surviving Sepsis Campaign: international guidelines for management of severe sepsis and septic shock, 2012. Crit Care Med.

[3] Garcia-Alvarez M, Marik P, Bellomo R (2014). Sepsis-associated hyperlactatemia. Crit Care.

[4] Hernandez G, Bruhn A, Castro R, Regueira T (2012). The holistic view on perfusion monitoring in septic shock. Curr Opinion Crit Care.

[5] Tapia P, Soto D, Bruhn A, Alegria L, Jarufe N, Luengo C (2015). Impairment of exogenous lactate clearance in experimental hyperdynamic septic shock is not related to total liver hypoperfusion. Crit Care.

[6] Cecconi M, De Backer D, Antonelli M, Beale R, Bakker J, Hofer C (2014). Consensus on circulatory shock and hemodynamic monitoring. Task force of the European Society of Intensive Care Medicine. Intensive Care Med.

[7] Jansen TC, van Bommel J, Schoonderbeek FJ, Sleeswijk Visser SJ, van der Klooster JM (2010). Early lactate-guided therapy in intensive care unit patients: a multicenter, open-label, randomized controlled trial. Am J Respir Crit Care Med.

[8] Ospina-Tascon GA, Umana M, Bermudez W, Bautista-Rincon DF, Hernandez G, Bruhn A (2015). Combination of arterial lactate levels and venous-arterial CO_2_ to arterial-venous O_2_ content difference ratio as markers of resuscitation in patients with septic shock. Intensive Care Med.

[9] Hernandez G, Luengo C, Bruhn A, Kattan E, Friedman G, Ospina-Tascon GA (2014). When to stop septic shock resuscitation: clues from a dynamic perfusion monitoring. Ann Intensive Care.

[10] van Genderen ME, Engels N, van der Valk RJP, Lima A, Klijn E, Bakker J (2015). Early peripheral perfusion-guided fluid therapy in patients with septic shock. Am J Respir Crit Care Med.

[11] Rivers E, Nguyen B, Havstad S, Ressler J, Muzzin A, Knoblich B (2001). Early goal-directed therapy in the treatment of severe sepsis and septic shock. N Engl J Med.

[12] Vallée F, Vallet B, Mathe O, Parraguette J, Mari A, Silva S (2008). Central venous-to-arterial carbon dioxide difference: An additional target for goal-directed therapy in septic shock?. Intensive Care Med.

[13] Ospina-Tascón GA, Bautista-Rincón DF, Umaña M, Tafur JD, Gutiérrez A, García AF (2013). Persistently high venous-to-arterial carbon dioxide differences during early resuscitation are associated with poor outcomes in septic shock. Crit Care.

[14] Hernandez G, Pedreros C, Veas E, Bruhn A, Romero C, Rovegno M (2012). Evolution of peripheral vs metabolic perfusion parameters during septic shock resuscitation: a clinical-physiologic study. J Crit Care.

