# Supplementary figures and images for: A hypoperfusion context may aid to interpret hyperlactatemia in sepsis-3 septic shock patients: a proof-of-concept study
Source: Ann Intensive Care. 2017 Mar 9;7:29. doi: 10.1186/s13613-017-0253-x (PMC5344869; doi:10.1186/s13613-017-0253-x)

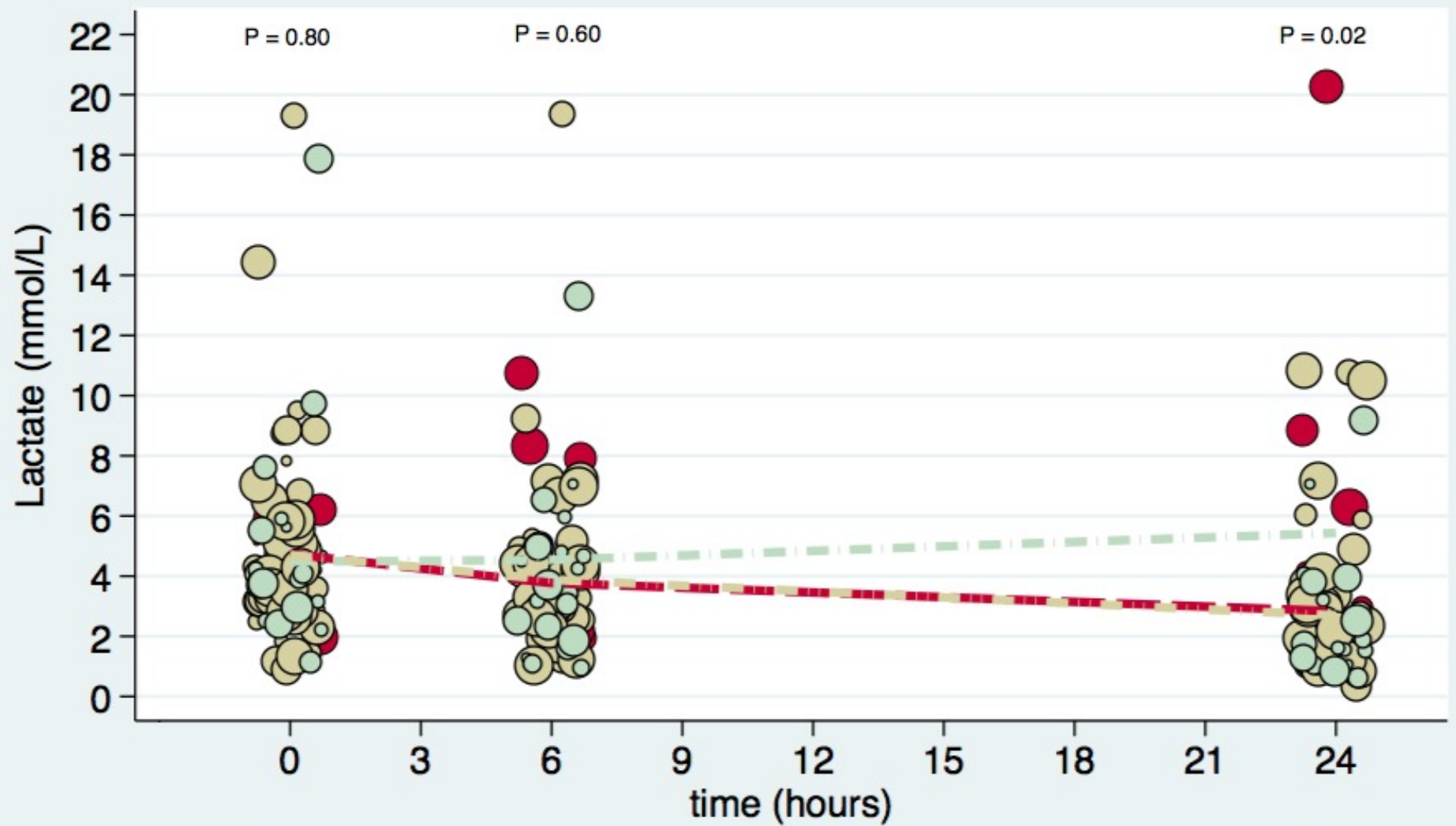

Supplement: Additional file 1. — Figure S1 Evolution of serum lactate levels over time (0, 6 and 24h) in different subgroups: patients without hypoperfusion context, and survivors and non-survivors in the hypoperfusion-context subgroup. [file 13613_2017_253_MOESM1_ESM.pdf]
