# Supplementary material for: A hypoperfusion context may aid to interpret hyperlactatemia in sepsis-3 septic shock patients: a proof-of-concept study
Source: Ann Intensive Care. 2017 Mar 9;7:29. doi: 10.1186/s13613-017-0253-x (PMC5344869; doi:10.1186/s13613-017-0253-x)
Supplement: Additional file 2. — Table S1 Clinical, demographic, severity scores, perfusion and hemodynamic variables at baseline for the whole cohort and according to different combinations of hypoperfusion criteria. A p < 0.05 was considered as significant. Values are expressed as mean ± SD. APACHE Acute Physiology and Chronic Health Evaluation, SOFA Sequential Organ Failure Assessment, P(cv-a)CO 2 central venous-arterial pCO2 gradient, ICU intensive care unit, LOS length of stay. [file 13613_2017_253_MOESM2_ESM.docx]

|  | All  Patients  (90) | Non  Hypoperfusion  related  (20) | Hypoperfusion-related | | | | | | | | | | | | | | | | |
| --- | --- | --- | --- | --- | --- | --- | --- | --- | --- | --- | --- | --- | --- | --- | --- | --- | --- | --- | --- |
|  |  |  | by  any criteria  (70) | p value | by SvO_2_  (21) | p value | by ΔCO_2_  (46) | p value | by CRT  (54) | p value | by SvO_2_ + ΔCO_2_  (17) | p value | by SvO_2_ + CRT  (15) | p value | by ΔCO_2_ + CRT  (31) | p value | all criteria  (12) | p value |  |
| Age (years) | 66 ± 16 | 65 ± 13 | 66 ± 17 | 0.9 | 68 ± 16 | 0.5 | 64 ± 17 | 0.8 | 65 ± 17 | 0.9 | 69 ± 16 | 0.5 | 66 ± 17 | 0.9 | 63 ± 17 | 0.5 | 67 ± 16 | 0.7 |  |
| Charlson Index | 1.8 ± 2.1 | 1.6 ± 0.3 | 1.9 ± 2.2 | 0.7 | 1.9 ± 2.2 | 0.7 | 2.0 ± 2.3 | 0.4 | 2.0 ± 2.3 | 0.4 | 2.1 ± 2.2 | 0.4 | 2.4 ± 2.3 | 0.3 | 2.2 ± 2.6 | 0.3 | 2.8 ± 2.3 | 0.2 |  |
| APACHE II score | 21 ± 7 | 20 ± 5 | 22 ± 7 | 0.2 | 21 ± 6 | 0.4 | 21 ± 7 | 0.4 | 21 ± 8 | 0.3 | 21 ± 6 | 0.4 | 21 ± 6 | 0.5 | 21 ± 7 | 0.6 | 21 ± 6 | 0.6 |  |
| SOFA score | 9.2 ± 3.5 | 8.4 ± 2.6 | 9.5 ± 3.8 | 0.1 | 8.9 ± 3.3 | 0.6 | 9.3 ± 3.9 | 0.3 | 9.6 ± 3.8 | 0.1 | 8.6 ± 3.3 | 0.8 | 9.3 ± 3.3 | 0.4 | 9.3 ± 3.8 | 0.4 | 9.0 ± 3.3 | 0.6 |  |
| Arterial lactate (mmol/L)  Baseline 0 h  6 h  24 h | 4.9 ± 3.1 | 4.7 ± 3.7  3.9 ± 2.7  2.7 ± 2.1 | 4.8 ± 2.8  4.0 ± 2.7  3.2 ± 3.0 | 0.9  0.9  0.4 | 5.1 ± 3.8  4.9 ± 4.0  3.5 ± 2.6 | 0.7  0.4  0.3 | 4.8 ± 3.2  4.1 ± 3.2  3.4 ± 3.5 | 0.9  0.8  0.3 | 5.0 ± 3.0  4.2 ± 2.9  3.5 ± 3.4 | 0.8  0.7  0.3 | 5.4 ± 4.2  5.2 ± 4.3  3.8 ± 2.8 | 0.6  0.3  0.2 | 5.3 ± 4.4  5.0 ± 4.4  3.8 ± 3.1 | 0.7  0.4  0.3 | 5.2 ± 3.7  4.5 ± 3.5  3.9 ± 4.1 | 0.6  0.5  0.2 | 5.5 ± 4.8  5.4 ± 4.8  4.2 ± 3.3 | 0.6  0.3  0.2 |  |
| Central venous O_2_ saturation (%) | 72 ± 9 | 79 ± 5 | 71 ± 9 | 0.001 | 62 ± 8 | <0.001 | 71 ± 10 | 0.01 | 71 ± 10 | 0.002 | 61 ± 9 | <0.001 | 61 ± 9 | <0.001 | 71 ± 11 | 0.002 | 61 ± 9 | <0.001 |  |
| P(cv-a)CO_2_ (mmHg) | 7.0 ± 2.8 | 3.4 ± 1.4 | 7.6 ± 2.6 | <0.001 | 7.8 ± 2.2 | <0.001 | 8.6 ± 1.8 | <0.001 | 7.4 ± 2.7 | <0.001 | 8.6 ± 1.8 | <0.001 | 7.4 ± 1.7 | <0.001 | 8.6 ± 1.7 | <0.001 | 8.0 ± 1.2 | <0.001 |  |
| Capillary refill time (s) | 4.6 ± 2.4 | 2.2 ± 0.4 | 5.3 ± 2.3 | <0.001 | 5.6 ± 2.3 | <0.001 | 4.8 ± 2.1 | <0.001 | 6.1 ± 2.0 | <0.001 | 5.5 ± 2.3 | <0.001 | 6.6 ± 2.0 | <0.001 | 5.8 ± 1.6 | <0.001 | 6.4 ± 1.9 | <0.001 |  |
| Central venous pressure at 6 h (mmHg) | 13 ± 5 | 14 ± 3 | 13 ± 5 | 0.3 | 12 ± 4 | 0.18 | 13 ± 4 | 0.4 | 13 ± 5 | 0.3 | 13 ± 3 | 0.3 | 12 ± 4 | 0.1 | 13 ± 3 | 0.3 | 12 ± 4 | 0.2 |  |
| Cardiac index (L/min/m^2^) | 3.2 ± 1.4 | 4.8 ± 1.8 | 2.9 ± 1.2 | 0.1 | 1.4 ± 0.2 | 0.22 | 3.1 ± 1.3 | 0.4 | 2.9 ± 1.4 | 0.3 |  |  | 1.5 ± 0.2 | 0.2 | 3.1 ± 1.9 | 0.4 |  |  |  |

|  | All  Patients  (90) | Non  Hypoperfusion  related  (20) | Hypoperfusion-related | | | | | | | | | | | | | | | | |
| --- | --- | --- | --- | --- | --- | --- | --- | --- | --- | --- | --- | --- | --- | --- | --- | --- | --- | --- | --- |
|  |  |  | by  any criteria  (70) | p value | by SvO_2_  (21) | p value | by ΔCO_2_  (46) | p value | by CRT  (54) | p value | by SvO_2_ + ΔCO_2_  (17) | p value | by SvO_2_ + CRT  (15) | p value | by ΔCO_2_ + CRT  (31) | p value | all criteria  (12) | p value |  |
| Mechanical ventilation (days) | 7.0 ± 6.9 | 5.2 ± 3.7 | 7.5 ± 7.5 | 0.06 | 6.5 ± 7.4 | 0.5 | 6.6 ± 6.6 | 0.2 | 6.9 ± 6.6 | 0.2 | 7.1 ± 8.1 | 0.4 | 5.6 ± 3.0 | 0.7 | 5.0 ± 2.9 | 0.9 | 5.6 ± 3.3 | 0.7 |  |
| ICU LOS (days) | 11.3 ± 9.1 | 9.1 ± 5.1 | 12 ± 9.9 | 0.08 | 11.3 ± 10.5 | 0.4 | 11.7 ± 10.4 | 0.2 | 11.6 ± 9.8 | 0.1 | 12.3 ± 12.0 | 0.3 | 10.8 ± 9.3 | 0.5 | 10.8 ± 10.5 | 0.4 | 11.5 ± 1.3 | 0.5 |  |
| Hospital LOS (days) | 24.5 (1-96) | 20.0 ± 10.0 | 26.0 ± 20.7 | 0.1 | 24.0 ± 19.1 | 0.5 | 25.5 ± 18.8 | 0.2 | 25.6 ± 21.5 | 0.2 | 26.7 ± 20.2 | 0.3 | 21.9 ± 15.5 | 0.8 | 23.8 ± 19.4 | 0.4 | 23.4 ± 16.9 | 0.6 |  |
| Hospital mortality (pts [%]) | 12 (13.5) | 1 (5) | 11 (16) | 0.2 | 4 (20) | 0.15 | 7 (16) | 0.23 | 8 (15) | 0.25 | 4 (25) | 0.09 | 3 (20) | 0.17 | 4 (13) | 0.35 | 3 (25) | 0.1 |  |
| Fluids 24 h (L) | 6,57 ± 2,56 | 5,94 ± 2,75 | 6,73 ± 2,52 | 0.2 | 6,93 ± 2,51 | 0.2 | 7,02 ± 2,73 | 0.2 | 7,03 ± 2,52 | 0.1 | 7,21 ± 2,37 | 0.2 | 7,48 ± 2,84 | 0.1 | 7,61 ± 2,74 | 0.04 | 7,8 ± 2,71 | 0.1 |  |
| Norepinephrine dose (ug/kg/min)  Baseline 0h  6h | 0.17 ± 0.20 | 0.09 ± 0.11  0.10 ± 0.10 | 0.19 ± 0.22  0.23 ± 0.25 | 0.04  0.03 | 0.16 ± 0.15  0.25 ± 0.31 | 0.1  0.05 | 0.18 ± 0.17  0.23 ± 0.25 | 0.03  0.006 | 0.21 ± 0.24  0.24 ± 0.27 | 0.005  0.002 | 0.15 ± 0.16  0.27 ± 0.34 | 0.24  0.07 | 0.19 ± 0.17  0.31 ± 0.35 | 0.08  0.05 | 0.19 ± 0.17  0.25 ± 0.27 | 0.02  0.01 | 0.17 ± 0.10  0.34 ± 0.39 | 0.2  0.07 |  |
| High-volume hemofiltration (pts [%]) | 17 (19) | 1 (5) | 16 (23) | 0.1 | 3 (14) | 0.34 | 10 (22) | 0.1 | 15 (28) | 0.04 | 3 (18) | 0.24 | 3 (20) | 0.19 | 9 (29) | 0.04 | 3 (25) | 0.11 |  |
| Use of inodilators (pts [%]) | 23 (26) | 1 (5) | 22 (31) | 0.01 | 5 (24) | 0.09 | 13 (28) | 0.03 | 18 (33) | 0.01 | 3 (18) | 0.22 | 5 (33) | 0.03 | 9 (29) | 0.04 | 3 (25) | 0.1 |  |

**Table S1** Clinical, demographic, severity scores, perfusion and hemodynamic variables at baseline for the whole cohort and according to different combinations of hypoperfusion criteria. A p< 0.05 was considered as significant. Values are expressed as mean ± SD. *APACHE*, Acute Physiology and Chronic Health Evaluation; *SOFA*, Sequential Organ Failure Assessment; *P(cv-a)CO_2_*, central venous-arterial pCO_2_ gradient; *ICU*, intensive care unit; *LOS*, length of stay.
